# Functionalised High-Performance Oxide Ceramics with Bone Morphogenic Protein 2 (BMP-2) Induced Ossification: An In Vivo Study

**DOI:** 10.3390/life12060866

**Published:** 2022-06-09

**Authors:** Filippo Migliorini, Jörg Eschweiler, Nicola Maffulli, Frank Hildebrand, Hanno Schenker

**Affiliations:** 1 Department of Orthopaedic, Trauma and Reconstructive Surgery, RWTH University Hospital, 52074 Aachen, Germany; migliorini.md@gmail.com (F.M.); joeschweiler@ukaachen.de (J.E.); fhildebrand@ukaachen.de (F.H.); hschenker@ukaachen.de (H.S.); 2 Department of Medicine, Surgery and Dentistry, University of Salerno, 84081 Baronissi, Italy; 3 School of Pharmacy and Bioengineering, Faculty of Medicine, Keele University, Stoke-on-Trent ST4 7QB, UK; 4 Centre for Sports and Exercise Medicine, Mile End Hospital, Barts and The London School of Medicine and Dentistry, Queen Mary University of London, London E1 4DG, UK

**Keywords:** high-performance oxide ceramics, alumina oxide, BMP-2, implantology, ossification

## Abstract

This study investigated the in vivo osseointegration potential of high-performance oxide ceramics (HPOCs) with peptide bone morphogenic protein 2 (BMP-2), comparing them with titanium implants. Histomorphometry was conducted around the distal, proximal, medial, and lateral sides of the implants to quantify the amount of mature and immature ossification within the bone interface. We hypothesised that HPOCs functionalised with BMP-2 promote ossification. HPOCs functionalised with BMP-2 were manufactured at the Department of Dental Materials Science and Biomaterial Research of the RWTH University Aachen, Germany. Histomorphometry was conducted by a professional pathologist in all samples. The region of interest (ROI) represented the percentage of the surrounding area of the implant. The percentages of ROI covered by osteoid implant contact (OIC) and mature bone–implant contact (BIC) were assessed. The surrounding presence of bone resorption, necrosis, and/or inflammation was quantitatively investigated. A total of 36 rabbits were used for the experiments. No bone resorption, necrosis, or inflammation was found in any sample. At the 12-week follow-up, the overall BIC was significantly increased (*p* < 0.0001). No improvement was evidenced in OIC (*p* = 0.6). At the 6-week follow-up, the overall OIC was greater in the BMP-2 compared to the titanium group (*p* = 0.002). The other endpoints of interest evidenced similarity between the two implants at various follow-up time points (*p* > 0.05). In conclusion, alumina HPOCs functionalised with peptide BMP-2 promote in vivo ossification in a similar fashion to titanium implants.

## 1. Introduction

Bone implants are common in orthopaedics and dentistry [1]. Several alloys are employed with different osseointegration capabilities. Osseointegration is defined as the direct contact of the implant with underlying bone under light microscopy [2,3]. Implant osseointegration is crucial to ensure implant survivorship. Titanium and its alloys are currently the best metals in terms of osseointegration; in contrast, given its absence of osseointegration, ceramic is biologically inert [4,5,6]. Ceramic implants have several advantages: high hardness and wear resistance, light weight, low modulus of elasticity and ductility, outstanding resistance to creep and compressive stress, and absence of artefacts in advanced imaging [7,8,9]. Titanium alloys may be responsible for hypersensitivity reactions, which may compromise implant longevity. When low-grade infection and other mechanical problems have been excluded, symptoms such as pruritus, pain, effusion, erythema, and hypersensitivity reactions should be taken into consideration [10]. Ions released by corrosion of metallic wear debris may impair ossification, and metal particles can be found in the soft tissues surrounding the implant [11]. Particles and ions may become clinically relevant for sensitive patients. According to the 2016 Australian Arthroplasty Register, approximately 2% of revision TKAs are attributed to metal-related pathology [12]. In selected patients with hypersensitivity, non-metallic implant solution as a possible savage revision is limited, with unpredictable results [13]. Current research to develop an alternative to metal alloys is ongoing. In this context, high-performance oxide ceramics (HPOCs) are attracting growing interest and broad research [14]. HPOCs provide strong wear resistance and excellent strength and are well cytocompatible, but are biologically inert. The biological inactivity of HPOCs, although reducing particle release and the risk of rejection and implant loosening, impairs the ossification of the implants in the surrounding bone tissue [4,5,6]. To overcome the lack of biocompatibility, we biologically functionalised their interface without altering the overall stability of the implants using the bone morphogenic protein 2 (BMP-2) [15,16]. Plasma enhanced chemical vapor deposition of silicon oxide (SiOx) was used to create an intermediate thin layer providing adhesion for BMP-2 [15,16]. BMP-2 stimulates the osteogenic differentiation of MSCs and osteoprogenitor cells into mature osteoblasts and supports bone formation in vivo [17,18,19]. However, evidence on the potential of ossification of BMP-2-coated implants is still limited. We hypothesised that BMP-2-functionalised HPOC implants promote ossification in vivo. To verify our hypothesis, the present animal study was conducted. This study investigated the osseointegration potential of HPOCs functionalised with peptide BMP-2 in 36 rabbits, comparing them with titanium implants. Histomorphometry was conducted around the distal, proximal, medial, and lateral sides of the implants to quantify the amount of mature and immature ossification within the bone–implant interface. 

## 2. Material and Methods

### 2.1. Sample Preparation

The HPOCs used for the experiments were manufactured at the Department of Materials Science and Biomaterial Research of the RWTH University Aachen, Germany. The process of obtaining the HPOCs is described in greater detail in previous studies [14,15,16,20,21,22,23,24]. Plasma-enhanced chemical vapor deposition (PE-CVD) was performed to facilitate the coupling of stable organosilane monolayers on the monolithic Al_2_O_3_ HPOC-based cylinders. These cylinders were activated using silicon suboxide (SiOx), which was deposited on the polished and cleaned Al_2_O_3_ HPOC-based cylinders. HPOC cylinders were then air-dried, cured at 80° for 45 min, and stored in liquid nitrogen until use. The day before surgery, HPOCs were coated with BMP-2. BMP-2 was purified using size exclusion centrifugation and coated over the functionalised HPOCs using bis(succinimidyl) suberate (BS3, Thermo Fisher, Waltham, MA, USA) crosslinkers as received. Purity was confirmed via antibody detection (monoclonal clone antibody anti-BMP-2, R&D Systems, Wiesbaden, Germany) and osteogenic induction capabilities via human mesenchymal stem cells (hMSC). Protein concentration was measured using a bicinchoninic acid assay (BCA, Thermo Fisher, Waltham, MA, USA). All experiments were performed in triplicate. Detailed information on the BMP-2 coating process can be found in our previous report [15,16]. In the control group, sandblasted titanium implants from Fa. Zimmer Biomet Deutschland GmbH (Neu-Ulm, Germany) with a diameter of 5.5 mm and length of 8 mm were used.

### 2.2. Surgical Procedure

This study was conducted according to the Animal Welfare Act of the Federal Republic of Germany. This study was approved by the Federal Office for Nature, Environment and Consumer Protection of North Rhine-Westphalia, Federal Republic of Germany (Approval ID: 84-02.04.2016.A434). For the study, 36 adult female New Zealand white rabbits with a minimum weight of 3 kg were used. Rabbits were randomly divided into four groups. For both implant types, 6- and 12-week groups were created. Before the surgical procedure, general anaesthesia was provided with 0.1 mL/mg/kg bodyweight medetomidine (Domitor) combined with 0.2 mL ketamine (Narketan) 10% via subcutaneous injection. The surgical site was shaved, disinfected with iodine and ethanol, and draped in a sterile fashion. Before incision, 10 mg/kg bodyweight enrofloxacin was injected subcutaneously. A skin incision was performed over the right lateral femoral condyle. After preparation through fascia and muscles, the condyle was exposed. The lateral collateral ligament (LCL) was identified as a landmark. Sparing the LCL, a mono-cortical drillhole with a 5.5 mm trephine was prepared under continuous irrigation to avoid thermal necrosis. After extraction of the bony cylinder, either a titanium or functionalised HPOC cylinder was inserted in a press-fit fashion. Attention was given to not produce lesions in the knee capsule. After irrigation with saline solution, tissues were closed in layers. Finally, the skin was stapled and sealed with chelated silver spray. For the first three days after surgery, 4 mg/kg bodyweight carprofen was applied every 24 h. Six and twelve weeks postoperatively, the animals were euthanized with 2 mL/kg bodyweight Natriumpentobarbital (160 mg Natriumpentobarbital/mL), and the femoral condyles were harvested.

### 2.3. Sample Preparation

The femoral condyles were harvested *en bloc*. Fixation was performed over 12 days with 4% paraformaldehyde followed by an alcohol series with ethanol of 50–100% and xylol. The specimens were embedded in Technovit 9100 (Fa. Heraeus Kulzer). Finally, coplanar thin cuts (60–70 µm) of the specimens were made with a diamond band saw (Exakt 300 CL). Grinding of titanium implants was performed with sandpaper, whereas functionalised HPOCs were ground with diamond paper. All specimens were stained with haematoxylin–eosin, trichrome, and toluidine. Histomorphometry was conducted by a professional pathologist with an OLYMPUS DSX-1000 digital microscope and stream desktop software (Olympus Hamburg, Germany). 

### 2.4. Histomorphometry

**Figure 1:**
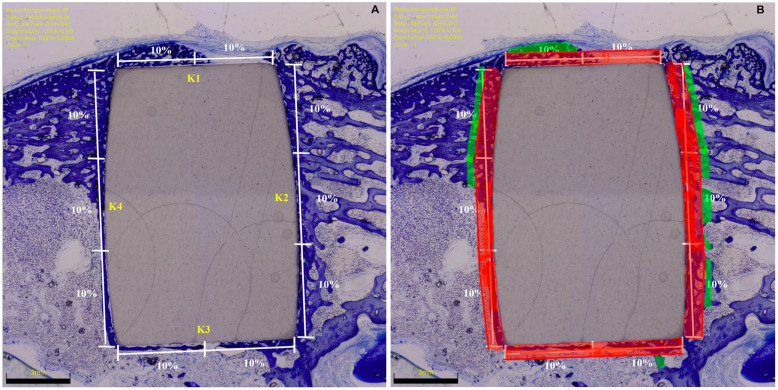
(**A**) Microscopical evaluation strategy of the BIC: K2 and K4 (longer sides) accounted for 60% (30% each), and the K1 and K3 (shorter sides) accounted for 40% (20% each). (**B**) Region of interest.

In the microscopical evaluation, implant sides were divided into four subsections: lateral (K1), distal (K2), medial (K3), and proximal (K4). The region of interest (ROI) represented the percentage of the surrounding area of the implant (green zones), which was analysed (red zones) (Figure 1). 

**Figure 2:**
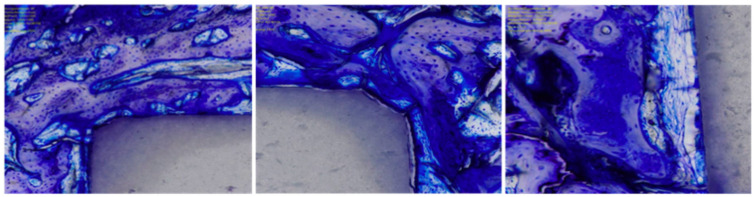
Functionalised HPOC implants (toluidine blue). Contour irregularities and porosities colonised by osteoblasts and osteocytes (left 150×; central 300×, right 400×).

**Figure 3:**
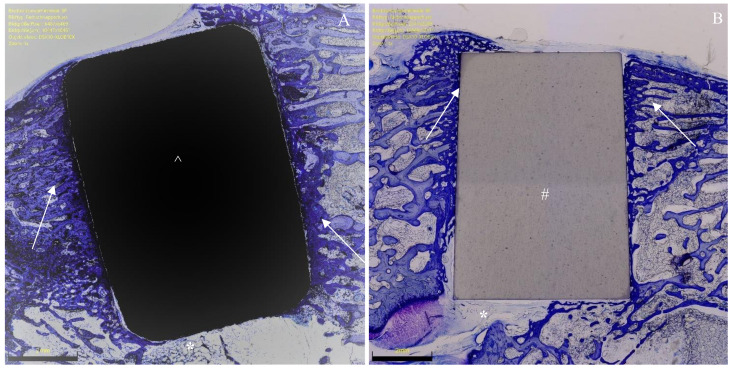
(**A**) Titanium implant (^) with surrounding bone formation (arrows) and soft tissue (*). (**B**) Ceramic implant (#) also with adjacent bone formation (arrows) and soft tissue (*). Section preparations stained with toluidine blue, each magnified 1×.

**Figure 4:**
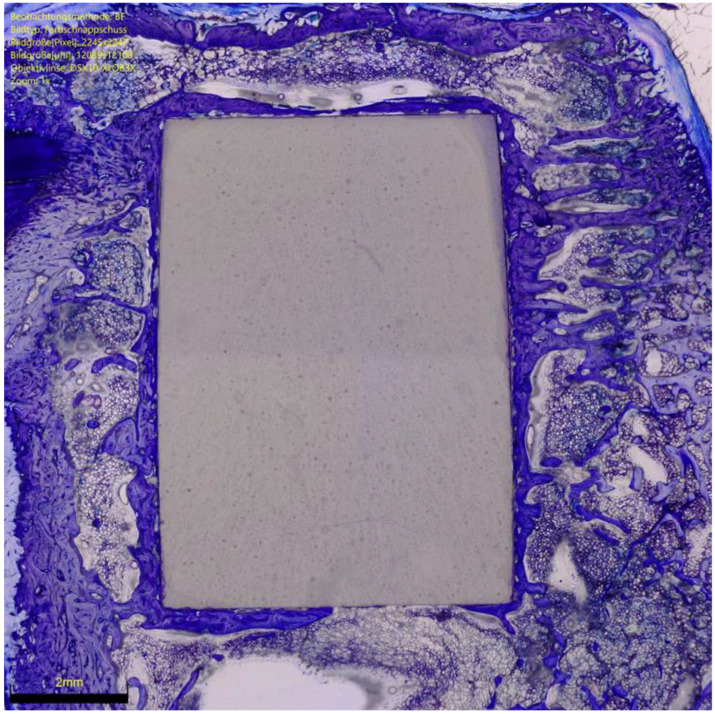
BMP-2-functionalised HPOC implants (toluidine blue). Little newly formed bone around the implant. Osteoid is focally detectable. Focal osteoclastic bone resorption.

Bone density was measured within each ROI by evaluating the percentage of area filled with mineralised bone. The percentage of ROI covered by newly formed, immature, and unmineralised bone matrix and the osteoid implant contact (OIC) were also quantified. Bone–implant contact (BIC) was assessed by analysing the length of mineralised bone with direct implant contact in percent. Surrounding presence of bone resorption, necrosis, and/or inflammation were quantitatively identified and classified: 0 (none), 1 (minimal), 2 (low), 3 (moderate), 4 (severe). Histomorphometry was conducted in the same fashion in all samples. Photos of histomorphometry are shown in Figure 2, Figure 3 and Figure 4.

### 2.5. Outcomes of Interest

The outcome of interest was to investigate the potential of osseointegration of BMP-2-coated HPOC implants in comparison to standard titanium implants. Hence, bone density; OIC; BIC; and the presence of bone resorption, necrosis, and/or inflammation were quantitatively assessed.

### 2.6. Statistical Analysis

The IBM SPSS (version 25) was used for the statistical analyses. For descriptive statistics, mean and standard deviation were calculated. For continuous data comparison, the mean difference effect measure was adopted, with standard error (SE) and *t*-value. The unpaired two-tailed T test was performed, with values of *p* < 0.05 considered statistically significant. The confidence interval (CI) was set at 95% in all the comparisons.

## 3. Results

### 3.1. Demographic Data

All 36 rabbits survived the 6- or 12-week experimental period. Six wounds had to be stapled again without any signs of wound infection or need for surgical revision. No rabbit died during the experimental period. At euthanasia, no clinical signs of inflammation or adverse tissue reactions were observed. All implants remained in situ. At baseline, rabbits had a mean weight of 3377.1 ± 286.4 mg. At last follow-up, rabbits had a mean weight of 3948.7 ± 383.2 mg. The mean weight difference from baseline was +571.6 mg (*p* < 0.0001). 

### 3.2. Ossification from 6 to 12 Weeks of Follow-Up

No necrosis, bone resorption, or inflammation was found in any sample. At 12-week follow-up, the overall BIC was statistically significantly increased (*p* < 0.0001). No improvement was evidenced in OIC (*p* = 0.6). These results are shown in greater detail in Table 1.

### 3.3. Comparison of BMP-2-Functionalised HPOCs versus Titanium Implants

At the 6-week follow-up, the overall OIC was greater in the BMP-2-functionalised HPOCs compared to the titanium group (*p* = 0.002). The other endpoints of interest evidenced similarity between the two implants at follow-up (*p* > 0.05). The results of the quantitative analyses are shown in greater detail in Table 2.

## 4. Discussion

The present study confirmed our hypothesis that alumina HPOCs functionalised with peptide BMP-2 promote in vivo ossification. The present in vivo investigation demonstrated that BMP-2-enhanced HPOC implants promote mature bone–implant contact from 6 to 12 weeks. Mature bone ossification was evidenced on the distal, proximal, and lateral sides of BMP-2-enhanced HPOC implant–bone interfaces. The longitudinal portions of cylinders (proximal and distal sides) are more subjected to load bearing, which represents an important growth factor [25]. The medial side of the cylinders, which is off load, evidenced the lowest ossification on implant–bone interfaces. Contrarily, on the lateral side of implants, although off load, greater ossification was evidenced. We assumed that the interruption of the continuity of cortical bone at the lateral side may have stimulated greater bone formation around the implant. The osteoid implant contact did not statistically significantly improve at 12 weeks. Concerning the comparison with standard titanium cylinders, functionalised HPOCs promoted similar ossification and implant integration to the titanium implants at 12 weeks. No necrosis, bone resorption, and inflammation were found in the samples at any follow-up.

Immature bone in the ROI during the first six weeks might results from the osteoinductive properties of BMP-2 and the earlier onset of bone regeneration triggered by BMP-2. Similar results were found by Hunziker et al. [26] in pigs, which demonstrated a higher osteoinductive potential in BMP-2-functionalised titanium implants. At 12 weeks, no difference between the titanium and BMP-2 group regarding osteoid regeneration was measurable [26].

Primary stability is still a concern when it considering early weight loading following surgical implants in musculoskeletal medicine [27]. Quicker osseointegration should favour the patient’s early recovery [28]. Given their biocompatibility, appropriate mechanical properties, and corrosion resistance, titanium and its alloys are commonly used [29,30]. Implant ossification has been extensively investigated; however, the rate of aseptic loosening and related complications (stress-shielding, persistent pain, inflammation) is still a concern [31,32]. Research on surface treatment is ongoing to improve osseointegration and soft tissue adhesion [33,34,35,36,37]; however, no consensus has been reached, and titanium alloys are preferred. Sandblasting or sandblasting combined with acid-etching has been proposed to enhance titanium implant osseointegration [38]. However, remaining sandblasting particles may negatively influence long-term stability [39]. BMP-2 has been used to enhance implant osseointegration in spine surgery [26,40]. Lan et al. demonstrated a higher quantity and better quality of osseointegration of human recombinant BMP-2-coated titanium implants in a rabbit model [41]. Moreover, human recombinant BMP-2 coating on titanium implants also improves bone healing and osseointegration in osteoporotic rats [42]. BMP-2 demonstrated an initiating and regulatory effect on osteoblasts [43]. Li et al. [44] evidenced promising results of titanium nanotubes functionalised with BMP-2 in osteoblast adhesion, proliferation, differentiation, and osseointegration. They also observed greater in vitro osseointegration and superior bone-bonding ability of BMP-2 compared to titanium implants [44].

Some important limitations of the present investigation must be acknowledged. Given the different appearance of titanium and HPOC implants, the pathologist and the surgeon were not blinded to the group allocation. This may increase the risk of detection and performance biases overestimating the results. Implant stability has not been mechanically tested, such as using a pull-out torque test. However, previous studies agreed that histomorphometry strongly correlated with pull-out torque tests [45,46,47,48]. A biomechanical evaluation of the mechanical properties of the formed bone, which could provide a comprehensive understanding of bone repair, was not conducted. Future investigations could benefit from longer follow-up. Between-species differences in biomechanics and gait in rabbits and humans and structural differences in anatomy and histology may limit the translational potential of our findings. However, being reproducible, low cost, and easy to handle, rabbit models are widely used.

## 5. Conclusions

The present study confirmed our hypothesis that alumina HPOCs functionalised with peptide BMP-2 promote in vivo ossification. The present in vivo investigation demonstrated that BMP-2-functionalised HPOCs promote bone–implant contact from 6 to 12 weeks. The osteoid implant contact did not statistically significantly improve at 12 weeks. Finally, functionalised BMP-2 HPOCs promoted similar ossification and implant integration to the titanium implants at 12 weeks. These results must be considered in light of the limitation of the present study.

## Figures and Tables

**Table 1:** Comparison of BIC and OIC of BMP-2-functionalised HPOCs at 6- and 12-week follow-up (SD: standard deviation; MD: mean difference).

Endpoint		6 Weeks	12 Weeks	MD	SE	95% CI	*t* Value	*p*
Lateral	BIC (%)	2.5 ± 2.0	4.4 ± 3.3	1.9	0.67	0.56 to 3.24	2.83	0.006
	OIC (%)	2.3 ± 2.3	2.4 ± 1.7	0.1	0.50	−0.90 to 1.10	0.20	0.8
Distal	BIC (%)	12.0 ± 6.4	15.6 ± 6.4	3.6	1.58	0.45 to 6.75	2.29	0.03
	OIC (%)	6.6 ± 4.7	6.0 ± 6.9	−0.6	1.45	−3.50 to 2.30	−0.41	0.7
Medial	BIC (%)	5.3 ± 3.5	6.1 ± 2.4	0.8	0.74	−0.68 to 2.28	1.08	0.3
	OIC (%)	2.6 ± 3.5	2.8 ± 3.6	−1.8	0.87	−3.55 to −0.05	−2.06	0.04
Proximal	BIC (%)	9.8 ± 7.0	13.8 ± 6.1	4.0	1.62	0.77 to 7.23	2.48	0.02
	OIC (%)	5.0 ± 0.0	5.0 ± 0.1	0.0	0.02	−0.04 to 0.04	0.00	0.99
Overall	BIC (%)	29.6 ± 5.1	39.9 ± 4.4	10.3	1.17	7.96 to 12.64	8.78	<0.0001
	OIC (%)	16.5 ± 1.2	16.2 ± 3.4	−0.3	0.63	−1.55 to 0.95	0.48	0.6

**Table 2:** Comparison of BMP-2-functionalised HPOCs versus titanium at 6- and 12-week follow-up (MD: mean difference). Negative mean difference indicates greater ossification in favour of the titanium group.

End Point		6 Weeks		12 Weeks	
		MD	*p*	MD	*p*
Lateral	BIC (%)	−0.6	0.4	0.6	0.4
	OIC (%)	2.3	0.007	2.0	0.005
Distal	BIC (%)	0.5	0.4	1.9	0.3
	OIC (%)	5.1	0.01	1.0	0.4
Medial	BIC (%)	−0.1	0.5	−3.1	0.009
	OIC (%)	2.9	0.02	1.3	0.2
Proximal	BIC (%)	−1.1	0.4	2.1	0.2
	OIC (%)	0.0	1.0	0.7	0.1
Overall	BIC (%)	0.0	0.4	0.1	0.2
	OIC (%)	10.2	0.002	5.1	0.1

## Data Availability

The data presented in this study are available on request from the corresponding author.
